# Sexual and reproductive health experiences, access to services, and sources of information among university students in Ethiopia

**DOI:** 10.3389/frph.2023.1271685

**Published:** 2023-12-14

**Authors:** Bekalu Mossie Chekol, Grace Sheehy, Yibeltal Siraneh

**Affiliations:** ^1^ Ipas Ethiopia, Addis Ababa, Ethiopia; ^2^ Ipas, Chapel Hill, NC, United States; ^3^ Faculty of Public Health, Institute of Health, Jimma University, Jimma, Ethiopia

**Keywords:** reproductive health, sexual health, health knowledge, health information, Ethiopia, university students

## Abstract

**Introduction:**

Adolescence and youth are times of major growth and change that can place young people at elevated risk of poor sexual and reproductive health (SRH) outcomes, particularly when they are living away from home for the first time. Understanding the barriers that young people face when accessing SRH services and information is imperative for addressing their SRH needs; our study explored this topic among university students in Ethiopia. Specifically, we explore university students' SRH experiences, access to services, and preferred sources of information.

**Methods:**

We draw on mixed-methods research conducted in four public universities across Ethiopia in early 2020. A random sample of 822 male and female students completed a cross-sectional survey that explored SRH knowledge, attitudes, experiences, and sources of information. We also conducted 8 focus group discussions with students and 8 key informant interviews across the four universities. Survey data were analyzed using descriptive statistics and we used structural coding and inductive analysis to analyze qualitative data.

**Results:**

The survey data demonstrated that condoms and emergency contraceptive pills were the most used contraceptive methods. Nearly 18% of female students had experienced an unwanted pregnancy, and 14% reported having had an abortion. Approximately one-third of students reported encountering gender-based violence in the past 6 months. Most students preferred receiving SRH information from mass media, and male students were more likely than female students to seek information from friends. Our qualitative findings highlight a lack of comprehensive SRH care available on university campuses, and services that are available are often of poor quality; participants described a lack of privacy, confidentiality and respect when accessing SRH care at school. University students also lack regular opportunities to learn about SRH while on campus, and their preferred sources of information varied widely.

**Discussion:**

Ethiopian university students have considerable unmet demand for SRH services and often face significant barriers to accessing high-quality information and services on campus. Consistent commitment and investment by universities and the government is vital to meeting the SRH needs of young people during this potentially vulnerable time.

## 1. Introduction

Adolescence and youth are periods of major physical, mental, and emotional growth and development (1). In addition to these physiological changes, social roles, responsibilities, and expectations also change (2). The unique vulnerabilities of this stage in the life course can place young people at elevated risk of unwanted pregnancy, sexually transmitted infections (STIs), HIV, and sexual violence (3). Many young people become sexually active during this time, and in some settings, it is common for women to begin childbearing during adolescence. In low-income countries, pregnancy and childbirth-related complications are the leading cause of death for adolescent girls (4). Sexual and reproductive health (SRH) experiences and needs change dramatically during this period, and young people often lack access to appropriate, non-judgmental SRH services and information by providers trained in the delivery of youth friendly SRH care. Access to appropriate, youth-friendly SRH services is often even more limited for youth living in low-resource settings (5). Due to pervasive stigma around adolescent sexuality and premarital sex, young people often experience judgement and stigma when seeking SRH services, which can further impede access.

The importance of meeting young people's SRH needs is particularly pronounced in low- and middle-income countries, where 90% of young people around the world reside (6). While efforts to meet the SRH needs of adolescents and youth have increased in recent decades, adolescent SRH remains a relatively new area of intervention in many countries in the Global South and many SRH programs continue to exclude or overlook this population (2). Despite these challenges, adolescence represents an important window of opportunity to set the stage for good health into adulthood (7), and meeting the health needs of young people is key to achieving the Sustainable Development Goals (8, 9). Meeting the SRH needs of young people requires more evidence on the state of their needs, experiences, and preferences.

In Ethiopia, adolescents and youth (ages 10–24) make up 33.8% of the population (10); the country has the second-largest population of youth in Africa (11). The Ethiopian government has made considerable investments and progress to advance SRH in recent decades, including among youth. The National Adolescent and Youth Reproductive Health Strategy aims to improve and expand youth-friendly health programming (12). However, adolescents and young people face challenges accessing SRH services and information: limited access to youth friendly SRH services, and rigid gender roles and social stigma around adolescent sexuality and premarital sex, all hinder access (13–17).

Research among young people in Ethiopia has found significant unmet SRH needs. Unmet need for family planning was estimated at 16% among all women ages 15–49 in 2019, and studies have found this number may be higher for adolescents and young women (18, 19). In 2016, the median age at first intercourse was 16 years old, with more than half of young people having intercourse before the age of 18 (20). Despite demonstrating familiarity with SRH matters, youth in Ethiopia often have low SRH service utilization; research among rural adolescents in east Gojjam zone found that 67% were knowledgeable about reproductive health topics like fertility, contraception and HIV, but only 21.5% had ever used reproductive health services (21). When young people begin university, they are often living away from home and their social support networks for the first time. They may also be newly exposed to substance use, sexual activity, peer pressure, and gender-based violence (22–24). Research from Haromaya University in Ethiopia found that one-fifth of undergraduate students had their sexual debut after beginning university (25). Nearly one-quarter of students surveyed from Ambo University reported experiencing an STI in the past year (26). University students often lack reliable access to information about use of highly effective methods of contraception, and research has found that use of emergency contraception among students is high (27, 28). Prior research among university and secondary school students in Ethiopia has found that discussing SRH with friends or family and having a health facility nearby are significantly associated with service utilization (29, 30). Many students rely on mass media for information on SRH (27), however, less research has explored preferred sources of SRH information among university students.

Understanding young people's needs and preferences for receiving SRH services and information is imperative for addressing their SRH needs, yet there are gaps in the evidence to inform programmatic efforts in this area. This study explored this topic among university students in Ethiopia, specifically, university students’ contraceptive use and experiences of unwanted pregnancy, abortion, and gender-based violence, as well as their access to SRH services on- and off-campus, and their preferred sources of information on SRH topics.

## 2. Methods

### 2.1. Study setting and overview

This study was conducted by Ipas Ethiopia in early 2020. Four public universities participated in a two-year pilot m-health intervention led by Ipas, which used SMS messages to provide tailored sexual and reproductive health information and referral services to students. The four universities included Dire Dawa University, located in the city of Dire Dawa; Arsi University, located in the city of Asella in Oromia State; Jimma University, located in Jimma City in Oromia State; and Dilla University, located in Dilla Town in the Southern Nations, Nationalities, and Peoples’ Region. This paper reports on mixed-methods baseline data from this pilot intervention. Baseline data assessed students’ knowledge, attitudes, and perceptions of SRH information and services, as well as the acceptability of m-health as a source of SRH information. A subset of these data is drawn upon from this study to explore students' SRH experiences, and access to SRH services and information while living in university campuses.

### 2.2. Study design and quantitative sample

The study design was a convergent mixed-methods design as part of a baseline study for the pilot intervention described above, which included both quantitative and qualitative methods, specifically surveys, focus group discussions and in-depth interviews. The sample for the quantitative survey was drawn using a stratified cluster sampling design. First, the sample was stratified by the four universities, and a cluster sampling approach was used within each university stratum. The sample was allocated across the four universities using probability proportional to size. University departments were the primary sampling unit and were randomly selected at each university using probability proportional to size. The secondary sampling unit was university students. A list of all students in alphabetical order by first name for each department was obtained from the respective university registrars. A proportional number of students were selected from each department; male and female students in any year were eligible to participate. Male and female students enrolled in each department were then sampled randomly using systematic random sampling. The total number of students in the four universities was 47,873 at the time of the study. Assuming the proportion of students with knowledge of key SRH indicators was 50% (31, 32), and using a 95% confidence interval, a design effect of 2 and accounting for 10% non-response, we aimed to sample 839 students. The STATCALC function of Epi Info version 7 was used for this calculation. Our final sample included 193 students from Dire Dawa University, 84 students from Arsi University, 344 students from Jimma University and 218 students from Dilla University. For this paper, we focused our sample of 822 students who had complete data on most of our primary variables of interest.

### 2.3. Quantitative data collection and analysis

Study team members coordinated with course instructors to distribute the surveys to selected students in their classrooms, and students were given time to complete and return the survey in class. Surveys were written in Amharic and were self-administered. The survey began by collecting some background information on the participant, including sex, age, and academic level. The remainder of the survey included questions on SRH knowledge, experiences and sources of information, and their opinion on the use of SMS text messages to provide SRH information.

Descriptive statistics were used to explore the sample's background characteristics, and the distribution of our quantitative variables of interest relating to SRH experiences and information. Primary outcome measures included sexual activity (any sexual activity in the past 6 months, yes/no); contraceptive use (any contraceptive use in the past 6 months, yes/no); abortion experience (ever had an abortion, yes/no); unwanted pregnancy (ever had an unwanted pregnancy, yes/no); experiences of GBV (encountered GBV in the past 6 months, yes/no); sources of SRH information used to learn about SRH (multiple answers allowed including friends, family, media, peer educators, etc.) and whether they discuss sex-related matters with family and/or friends (often/occasionally/never). Chi-square tests were used to examine differences in outcomes of interest, specifically relating to preferred sources of SRH information among female vs. male students. Survey data were analyzed using Stata 15.

### 2.4. Qualitative sample

We collected qualitative data via focus group discussions (FGDs) and key informant interviews (KIIs) at the same time as the survey data were collected in April 2020. The study team conducted two FGDs with purposively sampled students at each university: one with female students and one with male students, for a total of 8 FGDs across the four universities. Participants were recruited by m-health promoters working on campus, in collaboration with the University Gender Office and student representatives. The University Gender Office aims to enhance female students' academic experiences and empower female students. Each FGD included 6–10 students, and students who participated in FGDs did not participate in surveys. Facilitators used a semi-structured FGD guide to facilitate the discussions.

We conducted key informant interviews with two purposively sampled key informants per university who were knowledgeable about SRH issues and services, for a total of 8 key informants. Four key informants were student representatives and four were representatives of the university gender office. Interviewers used a semi-structured interview guide to facilitate the interview.

### 2.5. Qualitative data collection and analysis

All data were collected in Amharic, the local language in the study areas. FGDs explored norms and community perceptions of SRH, rather than more individual-level experiences. FGDs covered topics including SRH knowledge, experiences accessing SRH services and information on campus, barriers to SRH services, and preferred sources of SRH information.

Each key informant interview was facilitated by two trained interviewers, one who led the interview and the second who took notes. Data collectors were trained on the objectives of the study, how to use the discussion guide, facilitate the discussion, and take notes. All FGDs and interviews were audio-recorded with the consent of participants, and a research assistant transcribed recordings directly into English.

The first author (BMC) coded the FGD and KII transcripts using NVivo. After coding, he created matrices to help systematize analysis and summarize all the key categories. We applied structural coding based on priority topics and questions (33). We organized coded segments in a matrix using Microsoft Excel and further coded responses for data reduction and analysis. Through this process, we identified major themes focusing on barriers to SRH services and information and proposed solutions, which we organized into summary tables to guide the narrative. We used quotes from interviews and focus groups in this text to illustrate themes and show a range of perspectives. We also used our qualitative findings to increase the depth of our understanding of our quantitative results.

### 2.6. Ethical considerations

Ethical approval for this study was granted by Jimma University Institute of Health Ethics Approval Committee (IHRPGD/2095/2019). In addition, support letters were secured from Federal, regional, and local authorities for the lower-level government structures to provide the necessary support for the field data collection team. Informed verbal consent was obtained from each participant before the survey or interview began. Data collectors explained the study purpose and consent form to participants in their local language to ensure clear understanding. All data were kept confidential and stored in a secure place accessible only by the research team.

## 3. Results

### 3.1. Sample characteristics

The quantitative sample included 822 students at four universities in Ethiopia. Just over half (52%) of our sample was female, and most (74%) were between the ages of 20–24, and in 2nd year of university or higher (59%) (Table 1). Our qualitative sample included 60 students who participated in focus group discussions, and 4 student council representatives and 4 University Gender Office representatives who participated in IDIs (Table 2).

**Table 1:** Background characteristics of quantitative sample.

Background characteristics	*N* = 822	
	%	#
Age		
19 and under	20.0	158
20–24	74.4	589
25+	5.7	45
Sex		
Male	48.3	397
Female	51.7	425
University		
Jimma	41.5	341
Dilla	26.6	219
Dire Dawa	21.8	179
Arsi	10.1	83
Academic year level		
1st year	41.4	339
2nd year and above	58.6	480

**Table 2:** Characteristics of qualitative sample.

In-depth interviews (*n* = 8)	One student council representative per university, total of 4 participants One University Gender Office representative per university, total of 4 participants
Focus group discussions with female students (*n* = 4)	One FGD per university, total of 4 FGDs with female students. Female students were in years 1–4 of university, with an age range of 19–28.
Focus group discussions with male students (*n* = 4)	One FGD per university, total of 4 FGDs with male students. Male students were in years 1–4 of university, with an age range of 19–28.

### 3.2. Quantitative findings

#### 3.2.1. SRH experiences

Among our sample of female and male university students, 28.5% reported being sexually active in the past six months, while 24.8% of female students reported using a short-acting contraceptive method and 4.6% reported using a long-acting method (Table 3). Of those who had used a contraceptive method in the past 6 months, condoms and emergency contraceptive pills were the most used methods, reported by 40.7% and 34.8% of female students, respectively. Just under one-fifth of female students (17.7%) had ever experienced an unwanted pregnancy, and 13.7% reported having previously had an abortion. Nearly one-third of students reported encountering gender-based violence in the past 6 months, with 56.2% stating they had reported it (Table 3).

**Table 3:** SRH experiences among university students in Ethiopia.

Sexual activity and pregnancy	*N* = 805	
	%	#
Sexually active in past 6 months		
Yes	28.5	229
No	71.6	576
Contraceptive use in past 6 months (*n* = 416)^a^		
Used a short-acting method (condom, pills)	24.8	103
Used a long-acting method (implant, IUD)	4.6	19
No contraceptive use	70.7	294
Methods of contraception used in last 6 months		
Emergency contraception	34.8	41
Oral contraceptive pill	22.9	27
Injectable	11.9	14
Condom	40.7	48
IUD	5.1	6
Implant	13.6	16
Other^a^	5.9	7
Abortion experiences	*N* = 416	
Ever had unwanted pregnancy		
Yes	17.7	72
No	82.3	335
Ever had an abortion		
Yes	13.7	57
No	86.3	359
Relationships and violence	*N* = 797	
Encountered GBV in the last 6 months		
Yes	31.7	253
No	68.3	544
Reported GBV if encountered		
Yes	56.2	140
No	43.8	109

Questions about contraception and abortion were only asked to female students. The remaining outcomes were assessed among both male and female students, and variation in the total *N* by measure is due to missingness.

^a^ Other contraceptive methods included various traditional, non-medical methods.

#### 3.2.2. Sources of SRH information

Most students in our sample preferred receiving SRH information from mass media (57.1%), followed by peer educators (28.2%) and close friends (26.8%) (Table 4). Disaggregating by sex, we found no significant differences in most preferred sources of information, except for close friends—30.7% of male students sought information about SRH services from friends compared to 23% of female students (*p* < 0.05).

**Table 4:** Sources of SRH information among male and female university students in Ethiopia.

	Total^a^		Female students		Male students		
Sources of information about SRH services^a^	%	#	%	#	%	#	*p*-value
Peer educator	28.2	201	31	112	25.3	89	0.09
M-health/ mobile phone	17.4	124	16.1	58	18.8	66	0.35
Close friends	26.8	191	**23**	**83**	**30** **.** **7**	**108**	**0** **.** **02**
Mass media	57.1	407	55.4	212	58.7	195	0.37
Parents	18.2	130	16.9	61	19.6	69	0.35
Siblings	11.2	80	10	36	12.5	44	0.29
Other relatives	8.1	58	8.6	31	7.7	27	0.65
University clubs or offices	17.5	125	18.3	66	16.8	59	0.59
Other	5.1	36	3.6	13	6.5	23	0.07
Total^b^		713		361		352	
Discusses sex-related topics with friends or family							
Often	21.5	165	18.1	73	25.3	92	**0** **.** **05**
Occasionally	40.3	309	42.2	170	38.3	139	
Never	38.1	292	39.7	160	36.4	132	
Total		766		403		363	
Uses mobile phone to access SRH information							
Daily	9.8	73	7.6	29	12.3	44	**0** **.** **002**
Weekly/biweekly	18.1	134	17.2	66	19.0	68	
Monthly	13.8	102	12.5	48	15.1	54	
Other	6.5	48	4.4	17	8.7	31	
Never	51.9	385	58.39	224	45.0	161	
Total		742		384		358	

^a^ Students were asked about each source of information as a separate question, so the rows do not add up to 100%. The other two outcomes in the table reference categorical variables, so the rows do add up to 100% and there is only one *p*-value per variable.

^b^ The denominator varies for different measures due to skip patterns and some random missingness in the data.

Bold values represent figures that are statistically significant at *p* < 0.05.

Many participants either occasionally (40.3%) or never (38.1%) spoke about sex-related topics with friends and family. While 25% of male students said they discussed these topics often, 18% of female students did (*p* = 0.05). Just over half (51.9%) of participants said they never used their mobile phone to access SRH information, while 9.8% used it daily and 18.1% used it weekly or biweekly to access SRH information. Again, we noted differences by sex, with 12.3% of male students using their phone daily to access SRH information compared to 7.6% of female students, and 45% of male students never using their mobile phone to access SRH information compared to 58% of female students (*p* < 0.01).

### 3.3. Qualitative findings

Our qualitative results are divided into five themes relating to students' access to SRH services and information on campus.

#### 3.3.1. Limited SRH services are available on-campus

Most participants described an overall lack of comprehensive SRH care on campus, with many students needing to leave campus and incur additional costs to access necessary care, including contraception and abortion. Participants described a limited selection of available SRH services on their campus; many of which offer only short-acting methods of contraception (primarily condoms and emergency contraception), and sexually transmitted infection (STI)-related services. Students described even this limited selection as being frequently unavailable, with regular stockouts of commodities such as oral contraceptive pills and emergency contraception.

*The most common services available in our student clinic center are condoms, [emergency contraception] pills, and STI services. We have Implanon and other family planning methods, but we have shortages of emergency and oral contraceptive pills though the demand from students exists. We do not have abortion services.—*Student representative from Dilla University

*There are frequent shortages of drugs in our [on-campus] clinic. The clinic has no legal license to purchase drugs.—*Representative from gender office at Dire Dewa University

Participants widely agreed that SRH service availability was poorer on-campus than off, and that the availability of SRH services was inconsistent and disproportionate to the level of demand:

*There are organizations working on SRH issues in an on-and-off manner, but their effort is little compared to the size of problem.—*Female student from Arsi University

*We did not hear about organizations who provide SRH services and information [at our university]. Though there are 10,000 youths vulnerable to SRH risks, the university clinic doesn’t provide condoms in a consistent manner. Dissemination of [reproductive] health information and education is poor—*Male student from Arsi University

On-campus clinics also face challenges when trying to improve availability of SRH information and services, with concerns that this would promote sex among students:

*There is much resistance when we distribute condoms for students; by saying that making condoms available to students, that this is a factor pushing students to use it—*Representative from gender office at Dilla University

Abortion services are not available in most university clinics, and students are referred to off-campus clinics for this service. This leads many students to seek unregulated and at times unsafe abortion services from private clinics. Private clinics respond to demand from university students by opening facilities near campuses and tailored toward students' needs and preferences for services, such as for emergency contraception and MA drugs:

*Most students seek abortion information and services from private clinics outside of the university. Private clinics know these gaps and immediately open [abortion services] around the university.—*Representative from gender office at Jimma University

*Most students use emergency contraception (EC) pills from the pharmacy [to prevent pregnancy]. Pharmacists know the demand and consider it a business. There are significant numbers of students repeatedly using EC.—*Representative from gender office at Dire Dewa University

#### 3.3.2. Students lack opportunities to learn about SRH on campus

University students face numerous challenges when attempting to seek SRH information while on-campus; many participants described the dearth of consistent on-campus opportunities for students to learn about. or receive information and counselling on, SRH. Student clubs were often the sole source of SRH-related information, but few provided this information regularly and many were currently defunct. Some key informants described offerings such as one-time information sessions on SRH topics with no follow-up, often led by NGOs: *Other organizations organize a one-time event and disappear* (Representative from gender office at Dilla University). There was a sense among participants that nobody had responsibility or accountability for ensuring students had access to SRH information:

*There is a gender office and student representative office on the campus, but they are not well organized and functional to address students' reproductive health problems. The university did not have a well-established system to provide reproductive health services and information and help students overcome their reproductive health issues. Students depend on peer advice and ethical teachings provided from churches located outside of the campus.*—Male student from Jimma University

Students explained that SRH messaging was rarely integrated into schoolwide events, such as welcome events at the start of the school year. Some students had not learned anything about SRH during their time on-campus, and said that they had better access to SRH information when they were in high school than at university. This could contribute to misperceptions around SRH spreading and affecting students' willingness to seek SRH services.

*There are no [SRH] awareness activities at our university. I am 3rd year student and during this time I did not encounter any awareness activities organized by the university or other body about SRH—*Female students from focus group at Dire Dewa University.

*There is no basic SRH information and services provided from the clinic. A huge gap is observed in the provision of SRH information and services. It should be evaluated, and corrective actions need to be taken. Misconceptions [about abortion and contraception] emanate from students' background, religion, culture, and peer pressure. Students do not want to use most of the FP [family planning] methods.—*Representative from gender office at Jimma University

The limited SRH information that is provided to students is rarely comprehensive. Students described the content of SRH information sessions as insufficient and the framing as stigmatizing and unfriendly, leaving many feeling disinterested and unaware of SRH services available on campus:

*The information provided in the university about SRH is not sufficient. The [current SRH] program is once a week and it is not comprehensive. There is a tendency to say why do you do such mistake rather to assist and solve your problem. There are many students who do not get SRH information. And there are also many students who do not want to get SRH information and counseling by considering we already know it.*—Female student from Jimma University

*There is a lack of SRH awareness creation activities organized for students by the university … I am not sure whether or not the [SRH] services exist on this campus. I have not been exposed to such information.—*Male student from Arsi University

There is also a lack of open discussion about SRH on-campus, including among peers, families and in classes. Several students in science majors described a lack of SRH content in their courses, even when it would be relevant. This absence of open discussion was described by many FGD participants:

*There is no open discussion among students on SRH issues, it is considered as shameful and uncivilized if you talk and raise issues on SRH like HIV and STI among youth.—*Female student from Dire Dawa University

*We do not openly discuss about SRH among peers and family members. Even students and teachers did not freely and openly discuss SRH in biology courses. The teaching, learning approach, the curriculum, the culture and being young are the main factors which hamper openly discussing SRH issues … it is considered as shameful and not modern if you talk of SRH issues like HIV and STI among youths. Most students laughed at me when I talk about SRH—*Female student from Dilla University

Challenges with technology can also inhibit access to SRH information. For example, some universities do not have Wi-Fi, which hinders the ability of students to access SRH information online. Finally, if there are opportunities to learn about SRH on campus, they may not be inclusive or youth friendly. For instance, students who do not speak the local language may miss out on mass media programs providing SRH information or lose interest if the messaging is not engaging. Male students are also excluded from some educational outreach efforts; some campaigns specifically target female students with consequences for male students:

*This is one of its gaps- male students are neglected*—Gender office representative from Jimma University

*There is no organized way of delivering SRH information for both male and female students*. *There have been occasional events on campus to entertain students, and organizers have used these opportunities to educate students about their reproductive health, but they solely focused on female students.*—Male student from Jimma University.

#### 3.3.3. Available on-campus SRH information and services are often of poor quality

In addition to frequent stockouts of reproductive health commodities, students described overall poor quality of care for SRH services on-campus. Students felt that on-campus clinics often lacked the necessary supplies and equipment to provide comprehensive SRH services, and clinic staff lacked the necessary training and skills. Many students felt that their health-related concerns were not adequately addressed when they sought care on-campus, leading many to prefer off-campus SRH services:

*If a female student faces an unwanted pregnancy or HIV she will go to a clinic outside of the university. The health providers in the university clinic are not skilled, they are not willing to support students, but rather insult us. They do not want to give attention for students and no true counseling, diagnosis and examination is available. The clinic and lab have no equipment.—*Female student from Dire Dawa University.

*There are no skilled health workers working on reproductive health counseling and service in the campus clinic. Universities need to work on improving communication and competence of health care providers [in the clinic], they do not understand [our] problems. The service is poor, even non-existent—*Female student from Arsi University

In part, the dearth of skilled on-campus medical staff could be due to high staff turnover and variable levels of staff training, including on the provision of non-judgmental SRH care, an issue described by several participants as impeding the availability of services such as abortion:

*We had staff trained in abortion [care] but there is high turnover [of clinic staff]*– Representative from gender office at Dilla University.

*There is a large gap in the [reproductive health] services provided in the student clinic …Providers received [minimal] trainings on how to and when to provide SRH services with quality and compassion.*—Representative from gender office at Jimma University.

In addition to a lack of technical skills, participants also described long wait times, limited service availability, lack of proper supplies and equipment, low quality commodities (e.g., expired medicines), and poor management of patient information as further indicators of poor quality of care. Students described their experiences trying to obtain SRH services from on-campus clinics, and instead being offered a limited physical examination and an antibiotic or being encouraged to pursue abstinence instead of sexual activity.

*Students have complained [about] the service provided in the clinic and our office also had a discussion with health care providers to solve the problem. Most students say “the providers diagnosed us only for typhoid and typhus and provide the same drugs every time we visit the clinic. No other drugs are available in the clinic.” The clinic has no license to purchase medical drugs and it receives drugs from the hospital … Our office promotes abstinence for students until they graduate—*Student council representative from Dire Dawa University.

*There are no counseling referral linkage services in the campus to help students get the right counseling and information they need about their reproductive problems.*—Male student from Jimma University

*The university has [a] clinic, but it does not have condoms and does not provide information or education—*Male student from Arsi University

*Service providers do not properly listen and communicate with us, they are “kolie gefafi” [shocked when we seek SRH services and make us ashamed of it] and are not willing to provide SRH counseling and service. In addition, they seem to have low competency/low skill (usually they are interns)- they are medical students at practice and very few in number. Most students go and seek service from outside health facility in Assela town—*Female student from Dilla University

#### 3.3.4. Students lack privacy, confidentiality and respect when accessing SRH care

Another important dimension of quality care that is often lacking for students seeking SRH services on-campus is the provision of respectful care. Many participants described a lack of respect, privacy, and confidentiality serving as a deterrent to even attempting to seek SRH services on-campus. Providers were described as lacking bedside manners, insulting students who sought SRH services, and not respecting patient confidentiality. For some students, this meant seeking SRH services in private clinics off-campus.

*There is no privacy and confidentiality [during service provision], and no youth friendly services. As a result, students do not use the [reproductive health-related] services in the university clinic. Most students seek services from private clinics outside of the university or campus compound. … When students have STI problems, there is high fear of stigma to get the service in the clinic and as a result they prefer to use clinic outside of the university—*Representative from gender office at Jimma University.

*Students do not use abortion related services from university clinic to protect their confidentiality.—*Female student from Dilla University

SRH is heavily stigmatized, and students (especially female students) are often concerned about being seen seeking SRH care. The provision of SRH care by medical students both on-campus and in public health facilities near campus was a further deterrent to seeking care in these settings, as students were concerned about being recognized, facing judgment, and experiencing breaches in patient confidentiality by people they knew. This led to students incurring greater costs to travel further afield for SRH care.

*The hospital is near to the university and students do not want to use the services provided in the hospital due to medical students [from their university] who are working there—[there is] fear of stigma. Students who have money go to [private clinics] to get abortion services—*Representative from gender office at Dilla University.

Stigma surrounding abortion, pregnancy, contraceptive use, and STIs all further impede access to and use of SRH care, and students often travel away from campus to seek SRH services in a more private setting where they will not be recognized.

*If female students face pregnancy, she will first consult her boyfriend and best friend. She will not go to the university clinic due to fear of stigma. She wants to go to a health facility which is far from the university, even if she has no money, she will make her boyfriend bring money for the abortion.—*Female student from Jimma University.

#### 3.3.5. Students' preferred sources of SRH information and attitudes towards m-health are varied

University students turn towards various sources when seeking SRH information. Many students rely on their friends and family for information, while others seek information from reference books, textbooks, and mass media—however the accessibility and use of resources may be contingent on students' fluency in local languages or their ability to access mobile data or Wi-Fi:

*Some students went to their family, some watch educational videos, read newsletters use the library and internet to get the SRH information they need. However, as there is [usually] no internet connection, we are not always able to explore or browse information we would like to access—*Female student from Jimma University

Students from rural parts of the country may also face greater barriers to accessing SRH information, as they may be less familiar than their peers from urban areas with local sources of information and services:

*There is a large knowledge gap among freshmen students about SRH [compared to second year and above students]. The gap is high in all aspects of SRH including unwanted pregnancy, especially among those who come from rural areas. Students who come from urban areas know at least where to go and get the service if they face a problem—*Student council representative from Arsi University.

*National radio and television programs seem to have forgotten to discuss HIV and STIs. I guess prevalence of HIV is increasing since there are no or very limited youth friendly educational activities implemented in high schools and universities where greater numbers of adolescents and young girls are found. The media gives more attention to FP and maternal care, they do not provide adequate information about unwanted pregnancy, HIV and STI.—*Male student from Dilla university

The availability and depth of SRH information and knowledge also depended on the specific topic—several students mentioned that information on HIV was more easily accessible via mass media, and subsequently knowledge of this topic is better:

*We heard a lot about HIV from mainstream media and I think students have better information and knowledge about HIV than any other SRH issues—*Female student from Dire Dawa University.

When asked about m-health approaches to receiving SRH information, participants had mixed sentiments. Some thought this method would be appreciated by students because they could get the information they need without compromising their privacy:

*In one aspect, m-health technology or accessing SRH information in the form of text messages is a good idea. It will keep students' secrets and information confidentiality, as nobody can easily see the message from their personal cell phone. Other students will not able to see what the student read—*Representative from gender office at Dire Dewa University.

*Messages to inform female and male students about SRH could be in the form of short social media content or text messages which could lead students to sources with more information—*Male student from Dire Dawa University.

Others expressed doubt about the acceptability and usefulness of m-health approaches, stating that students would prefer something more engaging than text messages:

*I do not think m-health or text message strategies will work for students because students need more attractive and fun approaches like talk shows, and discussion sessions which could have entertainment elements—*Student council representative from Dire Dawa University.

Some students were doubtful about the utility of receiving SRH information via text message since they already receive many text messages and may overlook educational messages. According to participants, to ensure that mobile-based SRH information campaigns receive their attention, the program or app should be promoted directly to students, so that they know not to ignore the messages. Some students also emphasized that the costs of receiving text messages should be addressed.

*To effectively implement text message strategy or the m-health application, there should be an orientation for students beforehand [to ensure it won't be] confused with other unknown or promotional text messages—*Female student from focus group at Arsi University

*For the m-health or text message app to be effective, there should be frequent promotional activities [within the university] before launching or piloting the program. Without adequate promotion it will not be utilized there might be a need to air on the mass media to announce it for students –*Representative from gender office at Jimma University

*I do not think m-health or a text message app is a good way to reach students [and educate about SRH]. This is because students do not usually give attention for the text messages—*Female student from Jimma University

Other participants suggested that working to strengthen other means of delivering SRH information on-campus, such as via university recreation centers or student clubs, would be a more worthwhile effort instead of pursuing m-health interventions.

## 4. Discussion

Our study aimed to explore SRH experiences, access to services and information among university students in Ethiopia. We find that students lack access to comprehensive SRH services and information on-campus, and face considerable logistical, social, and economic barriers to accessing necessary care and resources. Young people may face heightened SRH-related risks and may be particularly vulnerable when living on campus (23). Thus, ensuring this group has access to high-quality SRH services and information is paramount for protecting their sexual and reproductive health into adulthood, and ensuring they can realize their educational ambitions. While prior research has explored levels of SRH knowledge among youth and university students in Ethiopia and other sub-Saharan African countries, fewer studies have explored access to SRH services and information, including preferred sources of information. Our research fills this gap in the evidence and provides clear entry points for interventions to improve SRH services and information tailored to university students' needs and preferences.

Nearly one-third of students in our study (29%) reported being sexually active, and contraceptive users overwhelmingly used short-acting methods, including emergency contraception, condoms, and oral contraceptive pills, likely due to limited choice of contraceptive methods on campuses. These findings are similar to prior research in Ethiopia; a study among female students at Wolaita Sodo University found that 24% were sexually active (34), while a meta-analysis of studies on emergency contraceptive use among female university students in Ethiopia identified a pooled prevalence of 34.5% (35). Approximately one-fifth of respondents in our study reported ever experiencing an unwanted pregnancy, and 14% reported having had an abortion. These numbers are higher than past research; a recent meta-analysis identified a pooled prevalence of induced abortion among female university students at 5.1% (36), however the magnitude of induced abortion at universities in Ethiopia ranged from 2.8% to 18.8% across studies (37, 38).

We found that SRH information and services were limited at the four universities, and often considered by both students and key informants to be of poor quality. Concerns about stigma, privacy and confidentiality kept many students from even attempting to seek SRH care on or near campus, while those who did seek services often found they could not access the services or commodities of their choice due to stockouts or poorly trained providers. Our findings align with other research in the country; Muntean et al. found that providers' negative attitudes toward unmarried young people were a barrier to SRH service delivery (15), while several studies have identified lack of information, shame and stigma, lack of privacy, and poor service availability as factors impeding access to SRH services (39, 40). Young people from wealthier families and those with better geographic access to services have been found to be more likely to use SRH services (41); participants in our study similarly described economic and geographic barriers to SRH service utilization, with students who came from rural areas and students who could not afford to pay for SRH care elsewhere often facing greater barriers to access.

Access to services and information are highly related; we found that university students lacked both, and these gaps in access were mutually reinforcing, such that a lack of information impeded access to services even when those services were technically available. In a study of university students in Gondar, Ethiopia, SRH knowledge was found to be better among students who had access to SRH services on campus (32). While there were some opportunities to learn about SRH on campus in our study, these opportunities were rarely comprehensive or consistent, and were often not inclusive of male students. This left many students to rely on other sources of information, including trusted people in their lives. Yet nearly 40% of students in our study reported that they never spoke about SRH matters with friends or family. This finding is similar to research among female secondary school students in Northern Ethiopia, which found that 61% discussed SRH matters with their peers and 44% with their parents (42). Comfort or openness in speaking about SRH with friends and family could have implications for service utilization; when young people speak with trusted people in their lives about SRH, they are often more likely to use SRH services. For instance, in a review of research from Ethiopia, adolescents who ever discussed SRH with family or health workers were 3.6 times more likely to use SRH services (43). Thus, providing opportunities for students to have open discussions about SRH on- and off-campus could be important for increasing utilization of SRH services.

The government of Ethiopia has made significant investments to improve the health of young people, including establishing a youth-focused program, enacting policies and laws, establishing national strategies and guidelines, among other efforts (11). Despite these efforts, young people continue to face significant reproductive health challenges. We identified several recommendations for improving access to SRH services and information on university campuses, which could improve SRH outcomes for young people across the country. Our participants identified a lack of ownership and accountability for ensuring students have access to SRH services and information on campus; establishing a mandate to integrate SRH content into the existing curriculum where appropriate, and establishing a responsible body on each campus that has responsibility for ensuring services and information are available to students is an important step in this direction. Providers and facility staff working on campus need further training on the provision of high-quality, non-judgmental, and youth friendly SRH care, as well as their ethical duty to maintain patient privacy and confidentiality. Sustained commitment and improved funding are also needed to ensure on-campus clinics remain stocked with a range of essential SRH commodities and supplies, and that providers are trained in the provision of comprehensive SRH care.

In terms of improving SRH information, many students felt that one-time learning opportunities were inadequate for meeting their needs for information. Consistent and longer-term SRH programs provided on-campus could be beneficial for ensuring students have regular and reliable access to SRH information and follow-up when needed. Students were interested in more opportunities to learn about SRH through peer support groups and discussion sessions, student clubs, and integration of SRH content in university events and classes. This information must be inclusive of male students and students not fluent in local languages. We found that approximately half of students used their mobile phones to access SRH information; this could represent an opportunity for exploring use of mobile devices to share information via SMS or mobile apps. However, participants cautioned that this information could easily be ignored or overlooked by people accustomed to a deluge of messages on their phones unless regular promotional activities are employed. Language accessibility should also be prioritized in the design of mobile interventions.

### 4.1. Study limitations

Our study has several limitations. Data were collected from four universities in Ethiopia and may not be representative of students' experiences at other schools or in other parts of the country. Our data were collected in Amharic and translated to English for analysis, and it is possible some nuance was lost in translation. Data were collected in early 2020, prior to COVID-19, and some of these dynamics may have shifted (and likely worsened) in the time since the study was conducted, as studies have demonstrated changes to SRH service utilization in numerous parts of the country since 2020 (44). Given the increased use of digital technologies on university campuses during the pandemic, it is possible students would feel differently now about the use of mobile phones to receive SRH information than they did at the time of the study*.*

## 5. Conclusion

Our findings demonstrate that university students in Ethiopia have significant unmet demand for SRH services and may face significant barriers to accessing high-quality information and services on campus. Students described a lack of consistent, high-quality, confidential, and youth-friendly SRH service delivery on-campus, leading many to seek services off-campus. Many students desired more opportunities to learn about SRH while at university and were open to the prospect of m-health programs to share SRH information. Consistent commitment and investment by universities and the government is vital to meeting the SRH needs of young people during this potentially vulnerable time. Further research is needed on how to deliver effective interventions to improve SRH outcomes among university students, and how these interventions can be scaled and adapted to different contexts.

## Data Availability

The raw data supporting the conclusions of this article will be made available by the authors, without undue reservation.
